# Structural and Functional Characterization of PR-4 SUGARWINs From Sugarcaneand Their Role in Plant Defense

**DOI:** 10.3389/fpls.2018.01916

**Published:** 2019-01-07

**Authors:** Flávia P. Franco, Renata O. Dias, Danyelle Toyama, Flávio Henrique-Silva, Daniel S. Moura, Marcio C. Silva-Filho

**Affiliations:** ^1^Departamento de Genética, Escola Superior de Agricultura Luiz de Queiroz, Universidade de São Paulo, Piracicaba, Brazil; ^2^Departamento de Genética e Evolução, Universidade Federal de São Carlos, São Carlos, Brazil; ^3^Departamento de Ciências Biológicas, Escola Superior de Agricultura Luiz de Queiroz, Universidade de São Paulo, Piracicaba, Brazil

**Keywords:** sugarcane, BARWIN, *C. falcatum*, chitinase, chitosanase, RNase

## Abstract

SUGARWIN1 and 2 are defense proteins from sugarcane. Their gene expression is known to be induced in response to wound and *Diatraea saccharalis* damage. Although the recombinant SUGARWIN protein does not affect insect development, it promotes significant morphological and physiological changes in *Fusarium verticillioides* and *Colletotrichum falcatum*, which lead to fungal cell death via apoptosis. In this study, we deepen our understanding of the role of SUGARWINs in plant defense and the molecular mechanisms by which these proteins affect fungi by elucidating their molecular targets. Our results show that SUGARWINs play an important role in plant defense against opportunistic pathogens. We demonstrated that *SUGARWINs* are induced by *C. falcatum*, and the induction of SUGARWINs can vary among sugarcane varieties. The sugarcane variety exhibiting the highest level of *SUGARWIN* induction exhibited a considerable reduction in *C. falcatum* infection. Furthermore, SUGARWIN1 exhibited ribonuclease, chitosanase, and chitinase activity, whereas SUGARWIN2 exhibited only chitosanase activity. This variable enzymatic specificity seems to be the result of divergent amino acid composition within the substrate-binding site.

## Introduction

The plant defense system is under constant selective pressure to improve its response to pathogens and insect damage (Cui et al., 2002; Medeiros et al., 2016). Pathogen recognition by plants activates the host defense response, resulting in cell wall fortification via callose and lignin synthesis, the production of secondary metabolites such as phytoalexins that exhibit an antimicrobial effect, and the accumulation of pathogenesis-related proteins (PR proteins) (Pieterse and van Loon, 1999).

The pathogenesis-related protein-4 (PR-4) family is a group of proteins equipped with a BARWIN-like domain. This domain can be associated with a chitin-binding domain, also well known as the hevein-like domain. This association separates the family into PR4 classes I (with the hevein-like domain) and II (without the hevein-like domain) (Broekaert et al., 1990; Neuhaus et al., 1996).

BARWIN is a PR-4 protein induced in barley by wounding or pathogens (Hejgaard et al., 1992; Svensson et al., 1992). Homologs of BARWIN have been identified in several plants, including tobacco (Friedrich et al., 1991), tomato (Linthorst et al., 1991), Arabidopsis (Potter et al., 1993), wheat (Caruso et al., 1999), *Wasabia japonica* (Kiba et al., 2003), maize (Bravo et al., 2003), rice (Agrawal et al., 2003; Zhu et al., 2006), *Lycoris radiata* (Li et al., 2010), apple (Bai et al., 2013), cacao (Menezes et al., 2014), and pepper (Kim and Hwang, 2015). Our previous studies have identified two homologs of BARWIN in sugarcane: SUGARWIN1 and SUGARWIN2 (Medeiros et al., 2012).

In many plant species, homologs of the BARWIN protein are associated with the plant response to fungal infection and mechanical wounding (Linthorst et al., 1991; Hejgaard et al., 1992; Caruso et al., 1999; Agrawal et al., 2003; Bravo et al., 2003; Li et al., 2010; Souza et al., 2017). In addition to SUGARWIN, antifungal activity has been ascribed to BARWIN-like proteins found in barley (Hejgaard et al., 1992), *W. japonica* (Kiba et al., 2003), wheat (Caruso et al., 2001), maize (Bravo et al., 2003), rice (Zhu et al., 2006), apple (Bai et al., 2013), and cacao (Menezes et al., 2014).

Pathogenesis-related protein-4 proteins are classified as chitinases (Neuhaus et al., 1996; Van Loon and Van Strien, 1999); however, several studies have also reported RNase activity for BARWIN-like proteins (Bertini et al., 2009, 2012; Guevara-Morato et al., 2010; Bai et al., 2013; Huet et al., 2013; Menezes et al., 2014; Kim and Hwang, 2015). RNA-binding site have been described for WHEATWIN1 (Bertini et al., 2009) and CARWIN (Huet et al., 2013), showing two important histidine residues for RNase activity, one at position 11 and another at position 111 (Bertini et al., 2009). Antifungal DNase activity was also observed, together with RNase activity, for the *Capsicum chinense* PR-4 protein (Guevara-Morato et al., 2010) and the *Theobroma cacao* TcPR-4b protein (Menezes et al., 2014).

The SUGARWIN1 and SUGARWIN2 proteins (*sugar*cane *w*ound-*in*ducible proteins) are believed to be part of a defense mechanism against pathogenic fungi in sugarcane plants via an salicylic acid (SA)-independent and jasmonic acid (JA)-dependent pathway (Medeiros et al., 2012; Franco et al., 2014). They are secreted proteins, induced in response to mechanical wounding, *Diatraea saccharalis* attack and after methyl jasmonate treatment, but not after methyl salicylate treatment (Medeiros et al., 2012). Besides SUGARWIN2 being highly induced by *D. saccharalis*, the recombinant protein has no effect on insect development but triggers changes in the hyphal morphology of *Fusarium verticillioides* and *Colletotrichum falcatum*, including increased vacuolization, multiple points of fracture in the cell wall, and extensive leaking of intracellular material, leading to cell death (Medeiros et al., 2012; Franco et al., 2014). The effect of SUGARWIN2 is specific to sugarcane pathogenic fungi and is not observed in fungi such as *Saccharomyces cerevisiae* and *Aspergillus nidulans*, which are unrelated to sugarcane diseases (Franco et al., 2014). The symptoms previously observed in fungi hypha suggest an enzymatic activity of SUGARWINs in fungus cell wall.

The sugarcane borer *D. saccharalis* (F.) (Lepidoptera: Pyralidae) is the major problem in sugarcane fields in Brazil, resulting in direct and indirect damage. The indirect damage is caused by fungi infection, such as *C. falcatum* (Went) [perfect stage: *Glomerella tucumanensis* (Speg.) Arx and Muller] that can take advantage of the openings produced by sugarcane borer to infect the plant (Ogunwolu et al., 1991; Cheavegatti-Gianotto et al., 2011). The red rot caused by *C. falcatum* is one of the major sugarcane diseases in Brazil and worldwide. Its main damage is due to inversion of sucrose by pathogen-induced invertase, resulting in dried sugarcane stalks (Viswanathan and Samiyappan, 1999). In Brazil, usually, the presence of this insect and fungi are correlated (Cheavegatti-Gianotto et al., 2011; Franco et al., 2017); however, *C. falcatum* infestation in the absence of *D. saccharalis* has been reported in other countries, including India, Australia, Thailand, Fiji, and the United States, showing high levels of damages in sugarcane production due red rot disease (Singh and Singh, 1989).

The purpose of this study was to deepen our understanding of the role of SUGARWINs in plant defense, identifying gene induction by *C. falcatum*, and the molecular mechanisms by which these proteins affect fungi, elucidating their molecular targets and enzymatic activity against the three main PR4 substrates, DNA, RNA and chitin/chitosan. We demonstrated that SUGARWINs are induced by *C. falcatum* infection in sugarcane, and the induction of SUGARWINs can vary among sugarcane varieties. Furthermore, SUGARWIN1 exhibited ribonuclease, chitosanase and chitinase activity, whereas SUGARWIN2 exhibited only chitosanase activity. This variable enzymatic specificity seems to be the result of divergent amino acid composition within the substrate-binding site that was demonstrated by protein modeling and docking studies. Our results show that SUGARWINs play an important role in plant defense against opportunistic pathogens and can be important to red rot disease control.

## Materials and Methods

### Sugarcane Variety, Fungus and Insects

Several genotypes of sugarcane (*Saccharum officinarum* x *Saccharum spontaneum*, cv. SP80-3280, SP80-1842, SP89-1115 e SP81-3250) were obtained from the Centro de Tecnologia Canavieira, Piracicaba, SP, Brazil. One-eyed sugarcane seed sets were disinfected with 0.01% chlorine, planted using a commercial planting mix (Plantmax, Eucatex, São Paulo, Brazil), and cultivated in a greenhouse under natural conditions.

*Diatraea saccharalis* caterpillars were reared on an artificial diet (Pompermayer et al., 2003) and were maintained at 25 ± 4°C and 60 ± 10% relative humidity with a light phase of 14 h. *C. falcatum* isolates were cultivated in potato dextrose (Difco^TM^, Sparks, NV, United States) medium and maintained at 25°C.

### *SUGARWIN* Gene Induction in Different Varieties of Sugarcane

Forty-day-old plants from the sugarcane genotypes SP80-3280, SP80-1842, SP89-1115, and SP81-3250 were used to identify differences in *SUGARWIN* gene expression. The assay was performed according to a previously published protocol (Medeiros et al., 2012). Third instar *D. saccharalis* caterpillars were starved for 12 h and individually placed on sugarcane seedling stalks. Plant material was collected 48 h after larval entry into the stalk region from approximately two centimeters around the point of inoculation and was frozen immediately. The control plants were left undisturbed, and were collected at 0 and 48 h. Three biological replicates of six plantlets were used for each time point and treatment. Analysis of *SUGARWIN1* and *2* gene expression was performed as described in the next section, and the varieties exhibiting the greatest contrast in *SUGARWIN1* and *2* expression levels were selected for use in the *C. falcatum* quantification assay.

### RNA Isolation, cDNA Synthesis and qRT-PCR

Total RNA from the sugarcane tissue was isolated with TRIzol Reagent (Invitrogen, Carlsbad, CA, United States) according to the manufacturer’s instructions, followed by DNA removal by treatment with 2 units of RNase-free DNase I (Fermentas, Vilnius, Lithuania) for 20 min at 37°C. Then, the RNA was re-extracted with TRIzol Reagent to remove any trace of DNA or DNase. Total RNA samples were quantified using a NanoDrop 2000 (Thermo Fisher Scientific, Wilmington, DE, United States), and their quality was assessed by agarose gel electrophoresis. First-strand synthesis was performed using ImProm-II Reverse Transcriptase (Promega Corp., Madison, WI, United States) according to the manufacturer’s instructions.

Quantitative real-time PCR was performed using a StepOne^TM^ Real-Time PCR system (Applied Biosystems, Waltham, MA, United States) and Maxima^®^SYBR Green/ROX qPCR Master Mix (2×) (Fermentas, Vilnius, Lithuania). Gene-specific primers for *SUGARWIN1*, *SUGARWIN2* and Glyceraldehyde 3-phosphate dehydrogenase (GAPDH) were used as described in Medeiros et al. (2012), the primers for rRNA 25S was used as described in Iskandar et al. (2004). GAPDH and rRNA 25S were used as endogenous controls and exhibited the same pattern of regulation; therefore, GAPDH was chosen as the reference gene. The reference genes used in this work were re-validated under experimental conditions. The amplification efficiencies and statistical analysis of relative expression results were performed as described in Pfaffl et al. (2002) using REST 2008 software, with primers efficiencies ranging from 90 to 99%. A *t*-test was performed between treatments using the R Statistical Software (Foundation for Statistical Computing, Vienna, Austria), considering significance levels of *p* < 0.01.

### *Colletotrichum falcatum* Inoculation in Different Sugarcane Varieties

Sixty-day-old sugarcane varieties were inoculated with 1 × 10^5^ *C. falcatum* conidia. The seedlings remained in a moist chamber in a greenhouse for 12 h and then were maintained for 10 days under natural conditions without supplemental artificial light. The control plants were left undisturbed, and were collected at 0 h, in the beginning of the experiment, and after 10 days of experiment. Samples were collected from the stalk region. Three biological replicates of six plantlets were used for each time point and treatment. The plant material collected was immediately frozen in liquid nitrogen for later transport to the laboratory and processing or storage at −80°C. These sample were used for *SUGARWIN*s gene induction quantification by qRT-PCR, same as described in the previous section, and for fungus quantification, as described in the next section.

### Plasmid Standard Curve for *Colletotrichum falcatum* Quantification in Sugarcane Plants

A plasmid standard curve was used to quantify *C. falcatum* contamination in sugarcane. This methodology uses a plasmid standard curve to quantify the number of target gene copies per PCR reaction. The number of plasmid molecules was determined according to Applied Biosystems (Waltham, MA, United States) instructions, using the following equation: Copies/μL = L ⋅$\left(\frac{C}{m.N}\right)$, where L represents Avogadro’s constant (6.022 × 10^23^ molecules/mol), C is the concentration of DNA in g/μL, m is the molecular weight of one basis point of DNA (660 g/mol), and N is the size of the plasmid in base pairs.

The ITS (rDNA internal transcribed spacer) gene is widely used for *Colletroticum* spp identification (White et al., 1990; Martinez-Culebras et al., 2000; Talhinhas et al., 2002; Mishra and Behera, 2009) and was used to design qPCR specific primers for *C. falcatum* quantification. First, *C. falcatum* conidia were grown in liquid PD medium for 48 h at 25°C and 250 rpm. Mycelia were collected by vacuum filtration, and after appropriate drying, they were frozen in liquid nitrogen and then macerated. DNA extraction was performed according to the method described in Moller et al. (1992). The plasmid standard curve was constructed by ligating an ITS gene fragment from *C. falcatum* into the pCR2.1 commercial plasmid vector from the TA Cloning Kit (Invitrogen) according to the manufacturer’s instructions. The ITS sequence was obtained from NCBI (gene bank accession number EU554112.1), and specific primers were designed using the program *OligoPerfect^TM^ Designer* (Forward 5′-GATGAAGAACGCAGCGAAAT-3′ and Reverse 5′-AACGGATCTCTTGGTTCTGG-3′). Plasmid DNA extraction was performed using a Plasmid Midi Kit (Qiagen, Hilden, Germany) according to the manufacturer’s instructions and was quantified using a NanoDrop 2000 (Thermo Fisher Scientific, Wilmington, DE, United States). The plasmid was sequenced to confirm its transformation and fragment orientation. Reference plasmid DNA was diluted (1:5; 1:25; 1:125; 1:625; 1:3125) to obtain plasmid genome equivalents for standard curve analysis. Quantification of *C. falcatum* in sugarcane plants was performed with specific ITS primers and the Standard Curve method using a PCR StepOne^TM^ Real-Time PCR system (Applied Biosystems, Waltham, MA, United States) and Maxima^®^SYBR Green/ROX qPCR Master Mix (2×) (Fermentas). The standard curves consistently demonstrated correlation coefficients (*R*^2^) of 0.99 and PCR efficiencies ranging from 90 to 100% when analyzed using StepOne^TM^ Software, version 2.0 (Applied Biosystems, Waltham, MA, United States). A *t*-test was performed between treatments using the R Statistical Software (Foundation for Statistical Computing, Vienna, Austria), considering significance levels of *p* < 0.01.

### Sugarcane Genomic DNA Extraction

Sugarcane DNA extraction was performed according to the methods described in Aljanabi et al. (1993) and was followed by treatment with RNase for 1 h at 37°C. DNA extraction was performed a second time using only chloroform:isoamyl alcohol (24:1) to remove the residual phenol from the samples. Total DNA was quantified using a NanoDrop 2000 (Thermo Fisher Scientific, Wilmington, DE, United States), and the quality was assessed with agarose gel electrophoresis.

### Ribonuclease Activity Assay

Ribonuclease activity was assessed as described in Caporale et al. (2004) with few modifications. Total RNA was isolated from *C. falcatum* with TRIzol^®^Reagent (Invitrogen, Carlsbad, CA, United States) according to the manufacturer’s instructions. The RNase activity detection of recombinant SUGARWIN1 and SUGARWIN2 (Medeiros et al., 2012) was performed at room temperature using 3 μg of *C. falcatum* RNA and purified protein in amounts varying from 0.25 to 3 μg in 10 mM Tris–HCl, pH 7.5, with 10 mM imidazole and 5 mM NaCl, in a total volume of 12.5 μl. After 1 h of incubation, the results were observed on a 1% agarose gel. Heat-inactivated SUGARWIN1 and 2 were used as controls.

### Deoxyribonuclease Activity

The deoxyribonuclease activity assay was performed as described in Guevara-Morato et al. (2010) with few modifications. To determine the DNase activity of recombinant SUGARWIN1 and SUGARWIN2 (Medeiros et al., 2012), 3 μg of *C. falcatum* DNA was incubated with the purified protein (0.25 to 3 μg) in a total volume of 12.5 μl in the presence of 10 mM Tris–HCl, pH 7.5, with 10 mM imidazole and 5 mM NaCl, in either the presence or absence of 2.5 mM MgCl_2_, since cofactors are required by DNases (Sanchez-Pons and Vicient, 2013), for 1.5 h at room temperature. The results were observed on a 1% agarose gel.

### Chitosanase Activity

The protein samples were added to SDS–PAGE sample buffer and heated at 100°C for 15 min. The proteins were separated on a 10% polyacrylamide gel containing 0.01% glycol chitosan (Sigma-Aldrich, St. Louis, MO, United States). The gel was cut into two parts; the negative control was not immersed in a refolding buffer, and the other part of the gel was immersed in a refolding buffer (50 mM Tris–HCl pH 7.5, 1% Triton X-100) at 37°C overnight. The gel was washed with distilled water and then stained with 0.01% (w/v) calcofluor white M2R in 10 mM Tris–HCl (pH 7.5). After 5 min, the brightener solution was removed, and the gel was washed with distilled water. Protein activity was visualized by placing the gels on a UV transilluminator (Trudel and Asselin, 1989). The two parts of the gel were photographed together.

### Chitinase Activity

Glycol chitin substrate was obtained by acetylation of glycol chitosan according to Trudel and Asselin (Trudel and Asselin, 1989). Glycol chitosan (250 mg) was dissolved in 2 ml of 10% acetic acid by grinding in a mortar. The viscous solution was allowed to stand overnight at 22°C. Methanol (9 ml) was slowly added and the solution was vacuum filtered through a Whatman No. 4 filter paper. The filtrate was transferred into a beaker and 150 μl of acetic anhydride was added with magnetic stirring. The resulting gel was allowed to stand for 30 min at room temperature and then was cut into small pieces. The liquid extruding from the gel pieces was discarded. Gel pieces were transferred to a Becker, covered with methanol, and homogenized using a TissueRuptor (Qiagen, Hilden, Germany) for 4 min at top speed. This suspension was centrifuged at 27,000*g* for 15 min at 4°C. The gelatinous pellet was resuspended in about one volume of methanol, homogenized, and centrifuged as in the preceding step. The pellet was resuspended in distilled water (10 ml) and homogenized for 4 min. This was the final 1% (W/V) stock solution of glycol chitin. The chitinase activity assay was performed as described before to chitosanase activity assay, using the glycol-chitin as substrate.

### SUGARWIN Structure Prediction and Protein-Ligand Docking Analysis

Three-dimensional structure prediction of both SUGARWIN proteins was performed using MODELLER software v9.14 (Webb and Sali, 2014). For this analysis, the structure of a homologous *Carica papaya* BARWIN-like protein (CARWIN – PDB: 4JP7, 1.05 Å) (Huet et al., 2013) exhibiting 76 and 71% identity to SUGARWIN1 and SUGARWIN2, respectively, was used as a template. Quality assessment of the predicted structures was performed using PROCHECK software (Laskowski et al., 1993).

The putative mode of interaction between SUGARWINs and both substrates, glycol-chitosan and chitin, was predicted using Haddock2.2 webserver and AutoDock Vina v. 1.1.2 software. The glycol-chitosan and chitin structures were obtained from the PubChem database (Wang et al., 2012) (PubChem CID: 131636552 and 21252321). Ligand structures were prepared for docking using MarvinSketch software v. 18.5 (ChemAxon^1^) and PRODRG2 server (Schuttelkopf and van Aalten, 2004).

Haddock predictions were carried out using the Easy Interface of Haddock2.2 webserver (van Zundert et al., 2016), applying the active and passive interface residues predicted by the CPORT algorithm (de Vries and Bonvin, 2011). For AutoDock Vina predictions, protein and ligand structures were prepared for docking using AutoDockTools v1.5.6 (Morris et al., 2009). AutoDock analyses were conducted using a large grid box (60 × 60 × 60 grid points, with a grid spacing of 1 Å) established around the protein, allowing an unbiased search of the entire protein surface for the putative binding site. For each docking analysis, a total of 50 independent runs were performed, generating 500 putative ligand positions.

Protein-ligand contacts from the ten best complexes predicted for each docking analyses were estimated using UCSF Chimera v 1.12 (Pettersen et al., 2004). These structures were selected as those presenting the best haddock-score inside the top cluster and the greatest protein-ligand binding affinity, for Haddock and AutoDock Vina, respectively. All structures were visualized using UCSF Chimera v 1.12 (Pettersen et al., 2004). Protein-ligand interactions from the best complex predicted by Haddock for each analysis were also predicted and visualized using LigPlot+ software (Laskowski and Swindells, 2011).

### SUGARWIN1 Mutant Production

The ORF encoding the *SUGARWIN1* was used as a template to perform the desired mutation. The *Sugarwin1* mutated was amplified by PCR using the forward primer 5’-ATCTCGAGAAAAGACAGCAGGCGAGCAACGTTCGGGCGACGTACAACTACTACAACCCG-3’ [the underlined region indicates the mutation of the codon CAC to AAC, which results in amino acid replacement of Histidine11 by Asparagine11, *Sugarwin1*(H11N)], and the reverse primer was the AOX3′ (pPICZαA vector primer).

The PCR was performed with approximately 25 ng of *SUGARWIN1* without mutation; 200 μM of each dNTP (Promega, Madison, WI, United States); 0.5 U of Q5 High Fidelity DNA polymerase (New England Biolabs, Ipswich, MA, United States); 1× PCR buffer with MgCl_2_ (New England Biolabs); 10 pmol of each oligonucleotide; and sterile water to achieve a final volume of 25 μL. The amplification was performed in a T100 thermocycler (Bio-Rad, Hercules, CA, United States), using the following program: 1 cycle at 98°C for 30 s; 35 cycles at 98°C for 10 s, 50°C for 30 s, and 72°C for 15 s; and a final extension at 72°C for 2 min.

The amplification product was analyzed by electrophoresis on a 1% agarose gel and visualized under UV light after staining with ethidium bromide. The amplicon was purified from the gel using the QIAquick gel extraction kit (Qiagen, Hilden, Germany) according to the manufacturer’s instructions. The purified product was then visualized on a 1% agarose gel and quantified.

After amplification, the *Sugarwin1* Mut ORF was digested with XhoI restriction enzyme (Thermo Fisher Scientific, Waltham, MA, United States) and cloned into a pPICZαA vector (Thermo Fisher Scientific). *Escherichia coli* DH5α strain was used to transform the recombinant plasmid. The recombinant plasmid was sequenced in a MegaBACE 1000 Flexible system using the DYEnamic ET Dye Terminator Kit for MegaBACE (GE Healthcare, Little Chalfont, United Kingdom).

## Results

### The *SUGARWIN1* and *2* Genes Are Differentially Induced in Sugarcane Varieties Challenged by *Diatraea saccharalis* Caterpillar

Our previous work showed that SUGARWINs are induced by *D. saccharalis*, methyl jasmonate and wounds (Medeiros et al., 2012). In this work, we aimed to identify the influence of SUGARWINs in *C. falcatum* contamination in sugarcane; therefore, we selected sugarcane varieties with different patterns of *SUGARWIN* gene expression. We used our previous knowledge about *SUGARWIN* gene expression in plants under *D. saccharalis* attack (Medeiros et al., 2012) to select sugarcane varieties.

**FIGURE 1:**
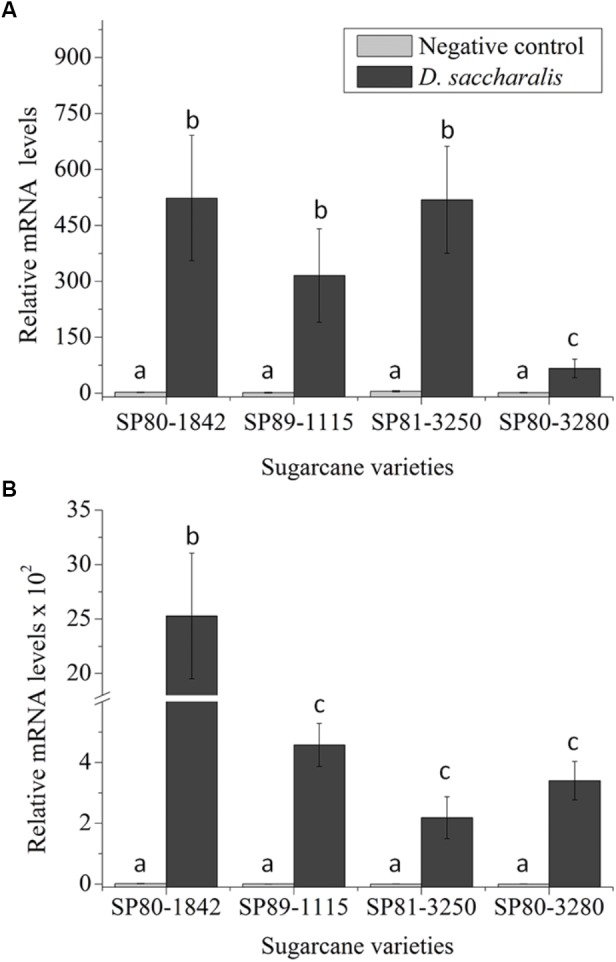
*SUGARWIN* gene expression in different sugarcane varieties. **(A)** *SUGARWIN1* and **(B)** *SUGARWIN2* gene expression after 48 h of *Diatraea saccharalis* infestation. Expression was quantified by qRT-PCR, and the values are presented as the mean (±SE) of transcript levels from three biological replicates normalized to the abundance of GAPDH. Gene expression was calculated using REST 2008 software (Pfaffl et al., 2002). Different letters indicate significant differences between treatments, as determined using a *t*-test (*p* < 0.01).

To evaluate *SUGARWIN1* and *SUGARWIN2* gene induction in different sugarcane varieties, we exposed SP80-3280, SP80-1842, SP89-1115, and SP81-3250 plants to *D. saccharalis* caterpillars. We observed differing levels of *SUGARWIN1* up-regulation in all varieties tested (Figure 1A). After 48 h of caterpillar attack, SP80-1842, SP89-1115, and SP81-3250 plants increased their *SUGARWIN1* mRNA levels in leaf stalks by up to 523, 315, and 518 times the levels found in the leaf stalks of 0 h control plants. The variety exhibiting the lowest mRNA induction level when attacked by *D. saccharalis* was SP80-3280, which increased its *SUGARWIN1* mRNA level approximately 66 times compared with 0 h control plants. The 48 h control plants showed no significant difference when compared to the 0 h control.

The *SUGARWIN2* gene was also up-regulated following *D. saccharalis* attack in all sugarcane varieties evaluated (Figure 1B). The increase in mRNA levels observed for the *SUGARWIN2* gene in SP80-1842 peaked at approximately 2.5 × 10^3^ times the levels found in the leaf stalks of 0 h control plants. The SP89-1115, SP81-3250, and SP80-3280 varieties exhibited low levels of *SUGARWIN2* mRNA induction following *D. saccharalis* attack, with 457, 218, and 340 times the 0 h control levels, respectively, the 48 h control plants showed no significant difference when compared to the 0 h control (Figure 1B). Based on the up-regulation of both *SUGARWIN* genes upon caterpillar attack, we selected the sugarcane varieties SP80-3280 and SP80-1842 as the low-SUGARWIN and high-SUGARWIN varieties, respectively.

### High- and Low-SUGARWIN Varieties Exhibit Differential Effects on *Colletotrichum falcatum* Infection

**FIGURE 2:**
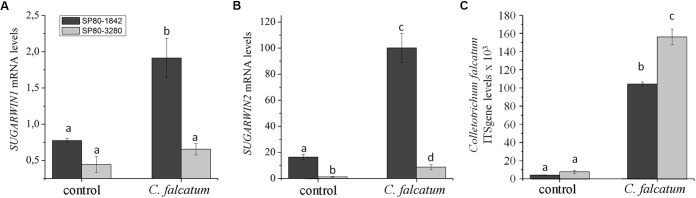
*SUGARWIN* gene expression and *C. falcatum* quantification in sugarcane. **(A)** *SUGARWIN1* and **(B)** *SUGARWIN2* gene expression after 10 days of treatment with *Colletotrichum falcatum* (*C. falcatum*) or without any treatment (control). Gene expression was quantified by qRT-PCR, and the values are presented as the mean (±SE) of transcript levels from three biological replicates normalized to the abundance of GAPDH. Regulation of expression was calculated using REST 2008 software (Pfaffl et al., 2002). Different letters indicate significant differences between treatments, as determined using a *t*-test (*p* < 0.01). **(C)** Quantification of *C. falcatum* was performed by qRT-PCR using the standard curve method and the ITS gene. The values are the mean (±SE) of three biological replicates. Different letters indicate significant differences between treatments, as determined using a *t*-test (*p* < 0.01).

Both high-SUGARWIN (SP80-1842) and low-SUGARWIN (SP80-3280) varieties were infected with *C. falcatum* fungus, and the levels of *SUGARWIN1* and *2* mRNA were evaluated. The sugarcane variety SP80-1842 exhibited 2- and 100-fold inductions in *SUGARWIN1* and *SUGARWIN2* mRNA levels, respectively, after 10 d of *C. falcatum* treatment when compared with the 0 h control (Figures 2A,B). The low-SUGARWIN variety exhibited 0.6- and 8-fold inductions in *SUGARWIN1* and *SUGARWIN2* mRNA, respectively, after 10 days of *C. falcatum* treatment, when compared with the 0 h control plants; however, the induction of *SUGARWIN1* in this sugarcane variety was not significantly different from the control (Figure 2A). The 10 d control plants showed no significant difference when compared to the 0 h control. To monitor the growth of the fungus, we also performed a *C. falcatum* quantification in infected plants and control plants. In the high-SUGARWIN, *C. falcatum* growth was approximately half that observed in the low-SUGARWIN variety (Figure 2C), showing an inverse correlation between *SUGARWIN* induction and *C. falcatum* infection.

### SUGARWIN1 and SUGARWIN2 Exhibit Differentiated Enzymatic Activities

**FIGURE 3:**
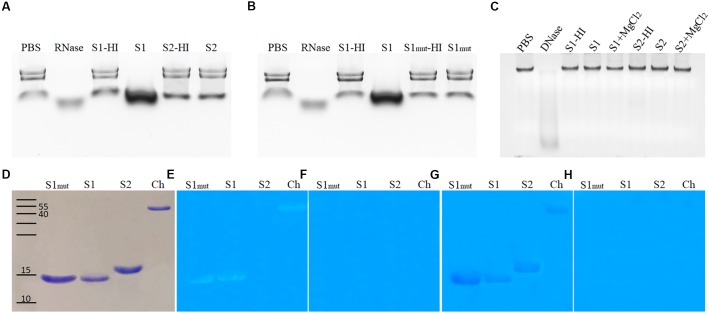
SUGARWIN1 and SUGARWIN2 enzymatic assay. **(A,B)** RNase assay. **(C)** DNase assay. The RNase assay was performed for 1 h and the DNase assay for 1.5 h. **(D)** SDS–PAGE stained with coomassie blue, **(E)** Chitinase assay, **(F)** Chitinase negative controls with denatured proteins, **(G)** Chitosanase assay, and **(H)** Chitosanase negative controls with denatured proteins. PBS, phosphate buffered saline (negative control); RNase, ribonuclease (positive control); S1-HI, SUGARWIN1-heat inactivated; S1, SUGARWIN1; S2-HI, SUGARWIN2-heat inactivated; S2, SUGARWIN2; S1mut-HI, SUGARWIN1(H11N) mutant-heat inactivated; S1mut, SUGARWIN1(H11N) mutant; DNase, desoxirribonuclease (positive control); S1+MgCl_2_, SUGARWIN1+ MgCl_2_; S2+MgCl_2_, SUGARWIN2+ MgCl_2_; Ch, chitinase (positive control).

To understand the SUGARWIN mechanism of action, recombinant SUGARWIN1 and 2 (Medeiros et al., 2012) proteins were used to perform enzymatic assays with typical PR4 substrates: RNA, DNA, chitin, and chitosan. Both chitin and chitosan are main constituents of the fungal cell wall (El Gueddari et al., 2002; Babu et al., 2015). To perform DNase and RNase assays, proteins were tested in different concentrations and over time. Only SUGARWIN1 was able to degrade RNA (Figure 3A) and an increase in activity was observed at higher protein concentrations and over time (data not shown). Buffer only or heat-inactivated protein did not show any change in RNA integrity. The SUGARWIN2 and SUGARWIN1(H11N) mutant proteins showed no RNase activity (Figures 3A,B). Neither SUGARWIN1 nor SUGARWIN2 was able to degrade DNA, even in the presence of MgCl_2_ cofactor (Figure 3C).

To test the chitinase and chitosanase activity of SUGARWINs, we used glycol-chitin and glycol-chitosan substrates, respectively (Figures 3D–H). Both SUGARWIN1 and SUGARWIN1(H11N) were able to degrade glycol-chitin, while SUGARWIN2 did not degrade glycol-chitin (Figure 3E). SUGARWIN1, SUGARWIN2, and SUGARWIN1(H11N) exhibited chitosanase activity (Figure 3G) and no activity was detected when proteins were heat-inactivated (Figures 3F,H).

### SUGARWIN Structural Characterization

**FIGURE 4:**
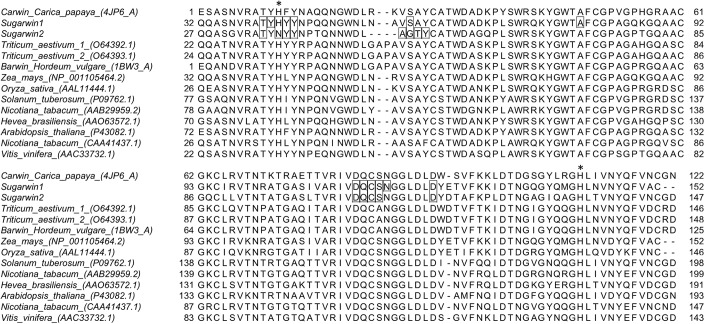
Sequence alignment for BARWIN homologous proteins from different plants. Binding-site residues predicted by docking analyses for SUGARWIN1 and 2 are highlighted in boxes. His11 and His111 positions are highlighted with an asterisk.

**FIGURE 5:**
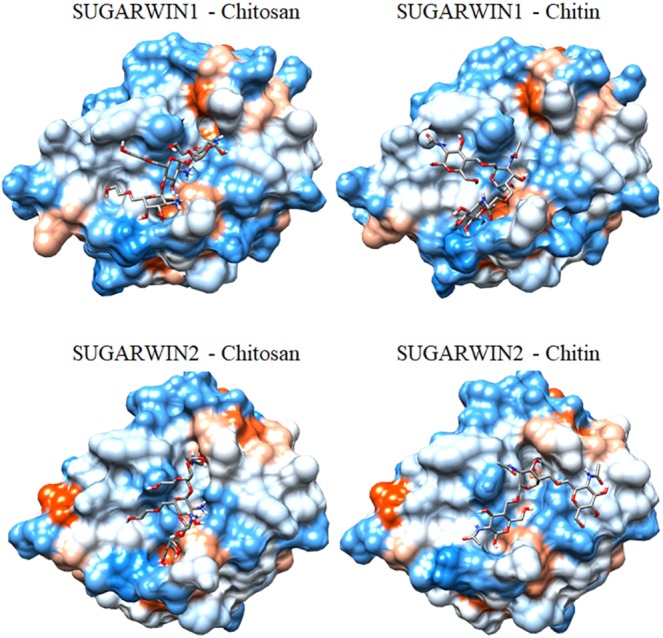
Putative mode of interaction between SUGARWINs and glycol-chitosan and chitin. The structures represent the best complex predicted by Haddock2.2 docking analysis. Protein surfaces are colored according the Kyte-Doolittle hydrophobicity scale, from blue for the most hydrophilic to orange for the most hydrophobic residues.

To understand the different substrate specificities observed during SUGARWINs activities analyses, the three-dimensional structure of both proteins was predicted by homology modeling. Several divergent amino acid positions were observed between SUGARWIN1 and SUGARWIN2 (Figure 4), some of which were located on the surface of the predicted structures. The involvement of these surface positions as putative ligand-binding sites was further analyzed using docking strategies (Figure 5).

Searches for glycol-chitosan- and chitin-binding sites in the predicted models produced similar results for both SUGARWINs. In order to improve the reliability of the prediction, binding-site residues for both proteins were selected as those contacting the ligand in more than five of the ten best complexes predicted for each analysis (Haddock and AutoDock Vina predictions, Supplementary Table S1). Furthermore, only binding-site amino acid positions identified by more than one analysis for each SUGARWIN are discussed below. Ten common amino acid positions (numbered according to the CARWIN template) – 9 (Thr), 11 (His/Asn), 12 (Tyr), 13 (Tyr), 26 (Ser/Gly), 82 (Asp), 83 (Gln), 84 (Cys), 85 (Ser), and 90 (Asp) – were contacted by glycol-chitosan and/or chitin in both proteins by bonded and non-bonded contacts (Figures 4, 5). These results highlighted the presence of a highly similar putative glycol-chitosan-/chitin-binding region in both SUGARWINs. However, some differences were observed in the docking results, with three amino acid positions exclusively contacted by ligands in SUGARWIN1 (Tyr10, Ala48, and Asn86), and three others in SUGARWIN2 (Ala25, Thr27, and Tyr28).

## Discussion

Plants are constantly being attacked by insects and pathogens and have developed sophisticated strategies to protect themselves. In a previous study, we identified two insect-induced genes homologous to *BARWIN* in sugarcane, called *SUGARWIN1* and *SUGARWIN2*. *SUGARWIN* genes are induced in sugarcane (SP80-3280) in response to insects, wounding and methyl jasmonate (Medeiros et al., 2012). However, the protein causes morphological and physiological changes in *C. falcatum* and *F. verticillioides* fungi (Medeiros et al., 2012; Franco et al., 2014). In this work, we found that *SUGARWIN* genes are induced at different levels by *D. saccharalis* depending on the sugarcane variety (Figure 1). Nevertheless, *SUGARWIN* genes are also induced by *C. falcatum* infection in the sugarcane varieties SP80-1842 and SP80-3280 (Figures 2A,B). Interestingly, the sugarcane variety SP80-1842, which exhibited high levels of *SUGARWIN* induction, was less susceptible to infection by *C. falcatum*, indicating that *SUGARWINs* could be linked to plant defense (Figure 2C). This pattern of plant response has been observed in other plants, such as *Pseudotsuga menziesii* (Islam et al., 2012), rice (Hao et al., 2009), and lentil (Mustafa et al., 2009). The varieties that show a pattern of high PR4 gene induction after pathogen infection show higher tolerance to the pathogen than varieties with low gene induction (Hao et al., 2009; Mustafa et al., 2009; Islam et al., 2012). CaPR4c, a BARWIN-like protein from pepper, was overexpressed in transgenic *Arabidopsis* plants and conferred greater resistance against pathogen infection (Kim and Hwang, 2015). *SUGARWIN* gene induction was lower in sugarcane plants when infected with *C. falcatum* than when infected with *D. saccharalis*. This can be result of a higher damage caused by the caterpillar, considering that *SUGARWIN* genes are also induced by mechanical wounding (Medeiros et al., 2012), and the mechanical damage caused by *C. falcatum* is lower when compared to *D. saccharalis* mechanical damage.

Gene expression studies have shown that the salicylic acid (SA) pathway is primarily activated in response to biotrophic pathogens, whereas the AJ and ET pathways are induced in response to necrotrophic pathogens (Epple et al., 1997; Staswick et al., 1998; Thomma et al., 1998; Vijayan et al., 1998). The PR genes can be induced by different pathways, the Arabidopsis pathogen-inducible genes PR-1, PR-2, and PR-5 are induced via a SA signaling pathway, whereas the plant defensin gene PDF1.2, thionin Thi2.7, PR-3 and PR-4 genes, are induced by pathogens via a SA-independent and JA-dependent pathway (Epple et al., 1997; Thomma et al., 1998). The infection by *C. falcatum* in sugarcane can activate several transcripts related to ethylene-mediated and jasmonic acid pathway of plant defense mechanisms (Prathima et al., 2013). As an hemibiotrophic pathogen, *C. falcatum* can show a sequential biotrophic and necrotrophic developmental stages (Muencha et al., 2008) and was able to induce PR4 *SUGARWIN* genes in sugarcane (Figure 2) which have also showed to be induced in a JA-dependent pathway (Medeiros et al., 2012).

The PR4 proteins are grouped into class I and class II based on the presence or absence of a chitin-biding domain (Neuhaus et al., 1996). These proteins are classified as chitinases due to the chitinase activity shown by a tobacco protein from the class I group (Ponstein et al., 1994). Others works revealed that PR4 proteins from class II, which show only a BARWIN domain, exhibit RNase activity (Bertini et al., 2009; Bai et al., 2013; Kim and Hwang, 2015). In some cases, the RNase and DNase activities occur in parallel (Guevara-Morato et al., 2010; Menezes et al., 2014). Cysteine proteinase inhibitor activity was also identified along with RNase activity in the pepper CaPR4c protein (Kim and Hwang, 2015).

SUGARWINs are homologs of PR4 class II (Medeiros et al., 2012); however, our enzymatic assays revealed that SUGARWIN1 exhibits RNase, chitinase and chitosanase activity (Figure 3), whereas SUGARWIN2 showed only chitosanase activity (Figure 3G). The enzymatic assays showed that protein folding is important for enzymatic activity, as denaturation by heating caused the loss of protein activity (Figure 3). DNase activity was not observed for either SUGARWIN1 or SUGARWIN2 (Figure 3C). Divalent metal cations are usually required by plant DNases (Sanchez-Pons and Vicient, 2013), however, even when in the presence of MgCl_2_ SUGARWINs showed do not affect DNA integrity.

**FIGURE 6:**
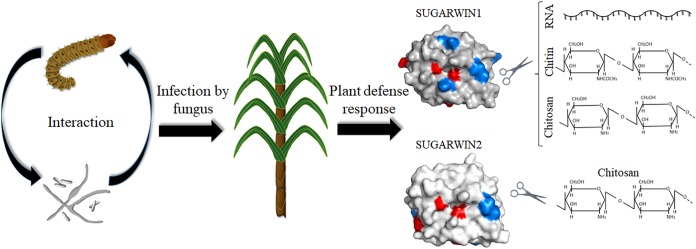
A summary of SUGARWINs enzymatic activity. The sugarcane borer *D. saccharalis* and the fungus *C. falcatum*, etiologic agent of red rot disease in sugarcane, can activate the induction of PR4 *SUGARWIN* genes in sugarcane. The product of these genes act as defense proteins affecting fungus morphology and viability due enzymatic activity. SUGARWIN1 shows RNase, chitinase, and chitosanase activity, whereas SUGARWIN2 shows chitosanase activity, as a result of different amino acid composition.

The main constituents of fungal cell wall are β1,3-glucan and chitins. The chitin can show differences in deacetylation degree (Feofilova, 2010), besides that, the chitosan, deacetylate chitin, can be present on the surface of the cell walls of fungal infection structures growing *in plant* (El Gueddari et al., 2002) indicating that chitosanases can represent a more efficient defense enzymes besides chitinases, or β1,3-glucanases (El Gueddari et al., 2002). The chitosanase activity of SUGARWIN2 described in this work corroborates our previous work, in which dramatic changes, including cell wall rupture, were identified in fungi after SUGARWIN2 treatment (Medeiros et al., 2012). These results differ from the results for other BARWIN-like proteins as WHEATWIN1, for example, which did not affect the fungal cell wall and exhibits only RNase activity (Bertini et al., 2009). These differences in enzymatic activity can be the result of variations in amino acid residues. The identified glycol-chitosan and chitin binding sites (Figure 5) has already been implicated in the RNase activity of other BARWINs (Bertini et al., 2009; Huet et al., 2013). Four amino acid positions contacted by glycol-chitosan and/or chitin in the docking analysis differ between SUGARWIN1 and SUGARWIN2: positions 11, 25, 26, and 27 are composed of His, Val, Ser and Ala in SUGARWIN1 and Asn, Ala, Gly and Thr in SUGARWIN2, respectively (Figure 4). These changes are capable of altering the binding site charge and shape and can be involved in SUGARWIN1 RNase and chitinase activity. A summary of SUGARWINs enzymatic activity are shown in Figure 6.

BARWIN RNase activity has been correlated with the presence of two highly conserved histidine residues: one at position 11 and another at position 111 (relative to the CARWIN structure) (Caporale et al., 2004; Bertini et al., 2009; Huet et al., 2013). Two different mutations in His11 (H11G and H11L) have been shown to partially inhibit RNase activity in WHEATWIN (Bertini et al., 2009), revealing the importance of this amino acid for RNase activity. Both SUGARWINs have the His111 residue; however, only SUGARWIN1 exhibits the His11 residue (Figure 4). The mutation of SUGARWIN1, changing the His11 to an Asn [SUGARWIN1(H11N)], showed loss of RNase activity suggesting that the ribonuclease activity of SUGARWIN1 may occurs according to the classical acid-base mechanism that involves two His residues, similar to RNase A, T1, and WHEATWINs (Caporale et al., 2004).

Our data shows that different sugarcane varieties have *SUGARWIN* genes with different levels of expression and this difference in mRNA accumulation may influence *C. falcatum* infection on sugarcane, reducing the damage caused by red rot disease in sugarcane. The antifungal activity of SUGARWIN proteins is likely due to a combination of their RNase, chitinase and chitosanase activity.

## Author Contributions

FF, RD, FH-S, DM, and MS-F designed the study and wrote the manuscript. FF, RD, and DT performed the experiments and analyzed the data. All authors read and approved the final manuscript.

## Conflict of Interest Statement

The authors declare that the research was conducted in the absence of any commercial or financial relationships that could be construed as a potential conflict of interest.

## Footnotes

1 http://www.chemaxon.com/products/marvin/marvinsketch/
